# The Real Importance of Pre-Existing Comorbidities on Long-Term Mortality after Acute Kidney Injury

**DOI:** 10.1371/journal.pone.0047746

**Published:** 2012-10-17

**Authors:** Mariana B. Pereira, Dirce M. T. Zanetta, Regina C. R. M. Abdulkader

**Affiliations:** 1 Division of Nephrology, School of Medicine, University of São Paulo, São Paulo, Brazil; 2 Department of Epidemiology, School of Public Health, University of São Paulo, São Paulo, Brazil; University of Sao Paulo Medical School, Brazil

## Abstract

**Background:**

The causes of death on long-term mortality after acute kidney injury (AKI) have not been well studied. The purpose of the study was to evaluate the role of comorbidities and the causes of death on the long-term mortality after AKI.

**Methodology/Principal Findings:**

We retrospectively studied 507 patients who experienced AKI in 2005–2006 and were discharged free from dialysis. In June 2008 (median: 21 months after AKI), we found that 193 (38%) patients had died. This mortality is much higher than the mortality of the population of São Paulo City, even after adjustment for age. A multiple survival analysis was performed using Cox proportional hazards regression model and showed that death was associated with Khan’s index indicating high risk [adjusted hazard ratio 2.54 (1.38–4.66)], chronic liver disease [1.93 (1.15–3.22)], admission to non-surgical ward [1.85 (1.30–2.61)] and a second AKI episode during the same hospitalization [1.74 (1.12–2.71)]. The AKI severity evaluated either by the worst stage reached during AKI (P = 0.20) or by the need for dialysis (P = 0.12) was not associated with death. The causes of death were identified by a death certificate in 85% of the non-survivors. Among those who died from circulatory system diseases (the main cause of death), 59% had already suffered from hypertension, 34% from diabetes, 47% from heart failure, 38% from coronary disease, and 66% had a glomerular filtration rate <60 previous to the AKI episode. Among those who died from neoplasms, 79% already had the disease previously.

**Conclusions:**

Among AKI survivors who were discharged free from dialysis the increased long-term mortality was associated with their pre-existing chronic conditions and not with the severity of the AKI episode. These findings suggest that these survivors should have a medical follow-up after hospital discharge and that all efforts should be made to control their comorbidities.

## Introduction

Recent studies have consistently shown that survivors of an acute kidney injury (AKI) episode have increased long-term mortality [1]–[3]. However, the risk factors associated with this increase in long-term mortality are controversial.

Some studies have shown that the most important risk factors for long-term mortality are the patient’s characteristics [1], [3]–[8]. Liaño *et al* followed 177 survivors of AKI and reported a mortality rate of 50% over a10 year period; the factors associated with the worst prognoses were advanced age, male gender, the presence of comorbidities and medical admission [1]. However, other studies have associated AKI characteristics with long-term mortality [3]–[7], [9]–[16]. Lafrance and Miller, reviewing the data from US veterans and following the patients for 2.3 years, showed that patients who suffered an AKI episode that did not require dialysis and who survived at least 90 days after the hospital discharge, had a relative risk of 1.41 of long-term mortality. This risk increased with increasing AKI severity [3]. In a systematic review, Coca *et al* found a mortality rate of 8.9 deaths/100 person-years for patients with AKI who survived hospitalization compared to 4.3 deaths/100 person-years for those without AKI who survived hospitalization. The risk was greater for patients with moderate and severe AKI. However, it is worth noting that the definition of AKI, as well as the length of follow-up, varied among the included studies [2].

To be able to propose interventions to decrease long-term mortality, it is important to identify the causes of death. It is known that the presence of chronic kidney disease (CKD) is a significant risk factor for the development of cardiovascular diseases and that the CKD population dies from these diseases [17]. However, it is not known whether cardiovascular diseases are also associated with long-term mortality after AKI. During the AKI episode, the causes of death are related to the clinical situation in which AKI develops, and the most important factor contributing to death is the underlying cause of AKI [18], [19]. Goldberg and Dennen noted that although several articles highlight the presence of previous comorbidities during the AKI episode, few investigate the influence of these comorbid illnesses on long-term mortality [20].

Moreover, different results can be found in developing countries because their populations may have different clinical features, different AKI characteristics and different AKI treatments [21]. Cerdá *et al* indicated that active research on the epidemiology of AKI in developing countries should be considered a priority [22].

Our objectives were to identify the ultimate causes of death among the survivors of an AKI episode and the influence of previous comorbidities on long-term mortality.

**Figure 1:**
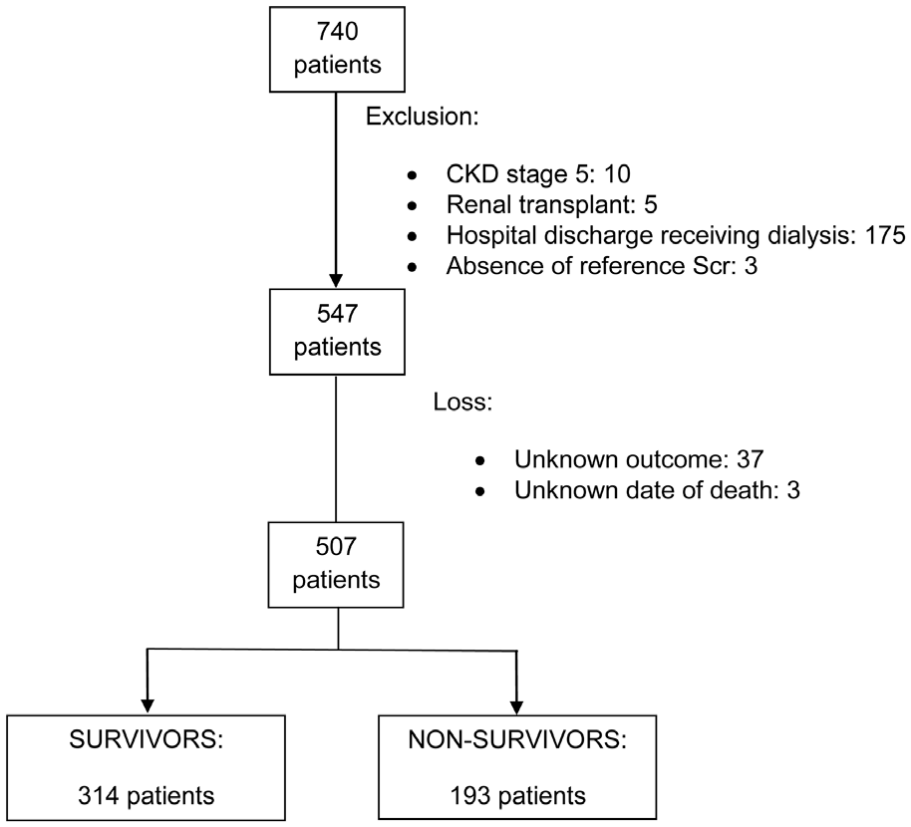
Flow-chart showing the selection process. CKD: chronic kidney disease Scr: serum creatinine.

## Materials and Methods

This is a retrospective study of patients hospitalized in two institutes of Hospital das Clínicas in São Paulo City (a general and a cardiologic hospital) with AKI diagnosis and followed by the same Nephrology Department. The study included AKI patients older than 17 years of age hospitalized from January 2005 to January 2007, who were discharged from hospital free from dialysis.

**Figure 2:**
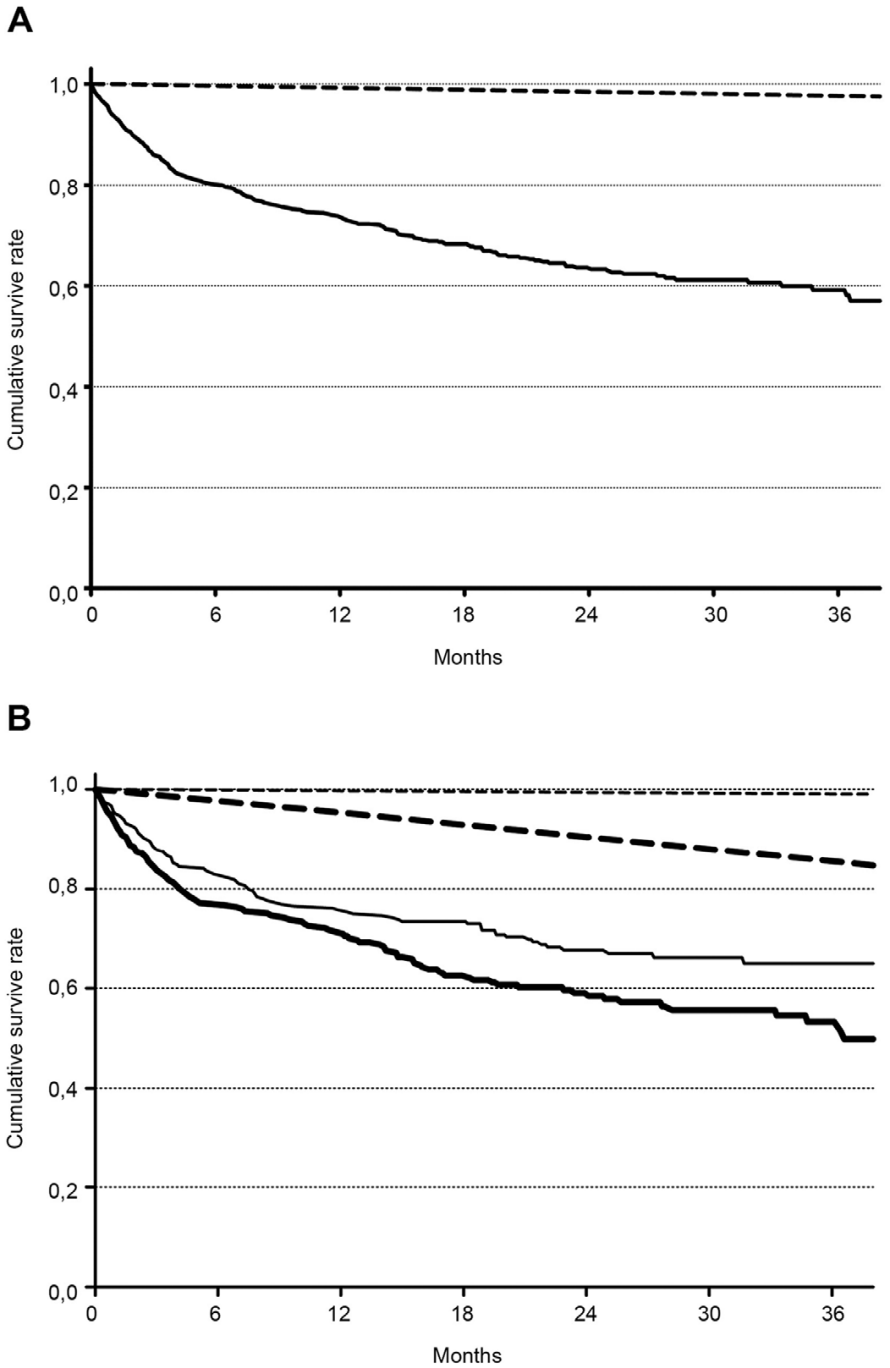
Kaplan-Meier survival curves of (a) the patients discharged alive after an AKI episode (solid line) and of the population of São Paulo city (dashed line); (b) Comparison of the effect of age on survival in patients discharged alive after AKI and in the population of São Paulo city. Solid lines represent the survival curves of AKI patients, and dashed lines represent the survival of the São Paulo population. Regular lines represent patients and population aged <65 years, and bold lines represent patients and population ≥65 years.

Patients with CKD stage 5 [glomerular filtration rate (GFR) <15 mL/min/1.73 m^2^ or reference serum creatinine (SCr) >3.5 mg/dL], kidney transplantation and without reference SCr were excluded. Patients discharged from the hospital receiving dialysis treatment were excluded. In order to study only patients with AKI caused by presumed acute tubular necrosis (ATN), those patients with AKI caused by glomerulopathies, multiple myeloma, urinary obstruction and obstetric conditions and those with less than two days of follow-up were also excluded.

**Table 1:** Characteristics of the included patients.

	All	Survivors	Non-survivors	P*
	N = 507	N = 314	N = 193	
*Male gender n* (%)	311 (61)	197 (63)	114 (59)	0.45
*Age ≥65 years n (%)*	266 (52)	135 (43)	106 (55)	0.01
*Comorbidities n (%)*				
Hypertension	287 (57)	178 (57)	109 (56)	1.00
Diabetes	166 (33)	100 (32)	66 (34)	0.62
Chronic liverdisease	44 (9)	21 (7)	23 (12)	0.05
Heart failure	108 (21)	55 (17)	53 (27)	0.01
Cancer	94 (18)	44 (14)	49 (25)	0.002
*Presumed CKD n (%)*	260 (51)	151 (48)	109 (56)	0.07
*Khan index n (%)*				<0.0001
Low risk	100 (20)	83 (26)	17 (9)	
Medium risk	195 (38)	125 (40)	70 (36)	
High risk	212 (42)	106 (34)	106 (55)	
*Type of admission* *n (%)* §				0.0025
Surgical	167 (33)	119 (38)	48 (25)	
Non-surgical	339 (67)	194 (62)	145 (75)	
*ICU admission* *n* (%)^□^	285 (58)	187 (61)	98 (53)	0.11

* Comparison between survivors and non-survivors, Fisher’s exact test or chi-square *n*,number of patients; CKD: chronic kidney disease; ICU, intensive care unit;

§ missing data in 1 patient

□missing data in 15 patients

### Definitions

AKI was defined at the first nephrology consultation as an absolute rise of 0.30 mg/dL or 50% in SCr achieved at that time relative to the reference SCr [3], [23]. Reference SCr was defined as the lowest SCr value found in the last 6 months before AKI or, for those without this measurement, the lowest value achieved during hospitalization in the absence of dialysis [24], [25]. GFR was estimated using the “Modification of Diet in Renal Disease” (MDRD) abbreviated equation [26]. A reference GFR <60 mL/min/1.73 m^2^ was considered as presumed CKD. Severity of AKI was classified into stage I, II or III according to the ratio between the highest SCr found during the AKI episode and the reference SCr: stage I ≤2, stage II 2 to 3, and stage III >3. All patients who needed dialysis were classified as stage III [3], [23]. AKI etiology was classified as ischemic (due to low cardiac output, hypovolemia, or hepato-renal syndrome), nephrotoxic (associated with drugs or heme pigments) or septic (sepsis or septic shock) [27]. Renal recovery was defined according to the ratio between the GFR at hospital discharge and the reference GFR: complete ≥90%; partial <90% and ≥50%; no recovery <50% [3]. Type of admission was classified as surgical or non-surgical according to the type of ward where the patient was admitted. The presence of the following pre-existing comorbidities was determined: hypertension, diabetes, heart failure, cancer, and chronic liver, coronary, pulmonary and vascular diseases. The patients were also stratified into three categories of risk using the Khan index; low risk: age <70 and no comorbidity; medium risk: age >70 and <80, or age <80 with one comorbidity; high risk: age >80, or any age with two or more comorbidities, or any age with malignancy [28], [29]. Other variables analyzed include age, gender, length of stay (LOS), length of Nephrology Department follow-up, intensive care unit (ICU) admission, need for dialysis, mechanical ventilation and vasoactive drugs and the presence of a second episode of AKI during the same hospitalization (defined as a new call for the nephrologist after a previous nephrological discharge).

**Table 2:** Characteristics of the acute kidney injury episode.

	All	Survivors	Non-survivors	P*
	N = 507	N = 314	N = 193	
*AKI etiology n (%)^&^*				
Ischemic	367 (73)	225 (72)	142 (75)	0.67
Septic	160 (32)	109 (35)	51 (27)	0.06
Nephrotoxic	149 (30)	101 (32)	48 (25)	0.08
*AKI severity n* (%)				0.20
stage I	135 (27)	81 (26)	54 (29)	
stage II	122 (24)	69 (22)	53 (27)	
stage III	250 (49)	164 (52)	86 (44)	
*Dialysis n (%)*	131 (26)	89 (28)	42 (22)	0.12
*Mechanical* *ventilation n (%)* ¥	161 (33)	115 (37)	46 (26)	0.001
*Vasoactive* *drug n (%)* §	205 (40)	136 (43)	69 (36)	0.09
*Recovery of renal* *function n. (%)*				0.87
No	70 (14)	42 (13)	28 (15)	
Partial	185 (36)	117 (37)	68 (35)	
Complete	252 (50)	155 (50)	97 (50)	
*Second AKI n. (%)*	40 (8)	16 (5)	24 (12)	0.004

* Comparison between survivors and non-survivors, Fisher’s exact test or chi-square.

AKI, acute kidney injury; *n*, number of patients;

& missing data in 7 patients.

¥ missing data in 21 patients.

§ missing data in 1 patient.

### Search for Long-term Outcome

The index date to determine the outcome was May31, 2008. The patients alive at this date were classified as “survivors”. The situation of each patient at the index date was obtained in the hospital database system by searching for either a report of death after hospital discharge (non-survivor) or for a medical consultation after the index date (survivor). We also consulted PRO-AIM, a register of death certificates in São Paulo City (PRO-AIM is the Portuguese abbreviation for Program for Improvement of Death Cause Information). In the case of patients for whom we were unable to obtain information, we attempted phone contact. Causes of death were identified on the death certificate obtained in PRO-AIM and were classified according to the codes of “International classification of diseases, 10th revision” (ICD-10). They were compared with the causes of death of the population of São Paulo City, which was also obtained in PRO-AIM [30].

**Figure 3:**
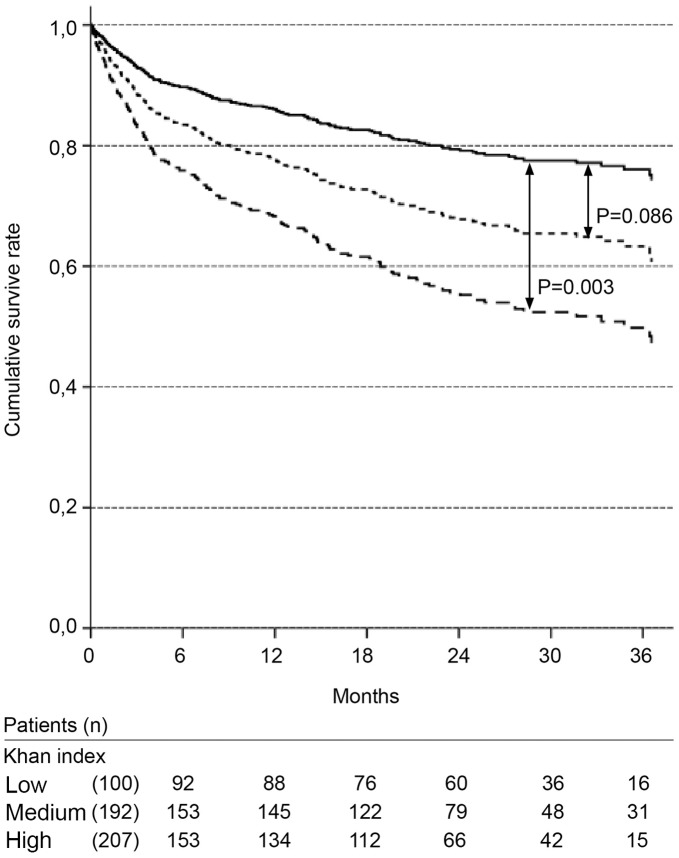
Cox survival curves according to Khan index. Solid line: low risk; dotted line: medium risk; dashed line: high risk. Adjusted for: gender; age; presence of cancer, heart failure, and presumed chronic kidney disease; nephrotoxic and septic etiologies.

**Figure 4:**
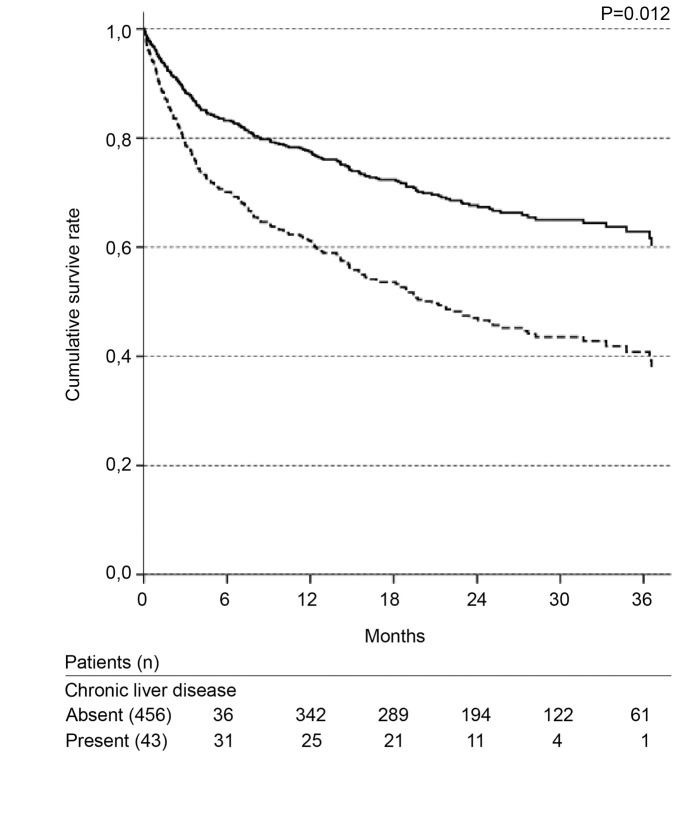
Cox survival curves according to chronic liver disease. Solid line: absence; dotted line: presence. Adjusted for: gender; age; presence of cancer, heart failure, and presumed chronic kidney disease; nephrotoxic and septic etiologies.

**Figure 5:**
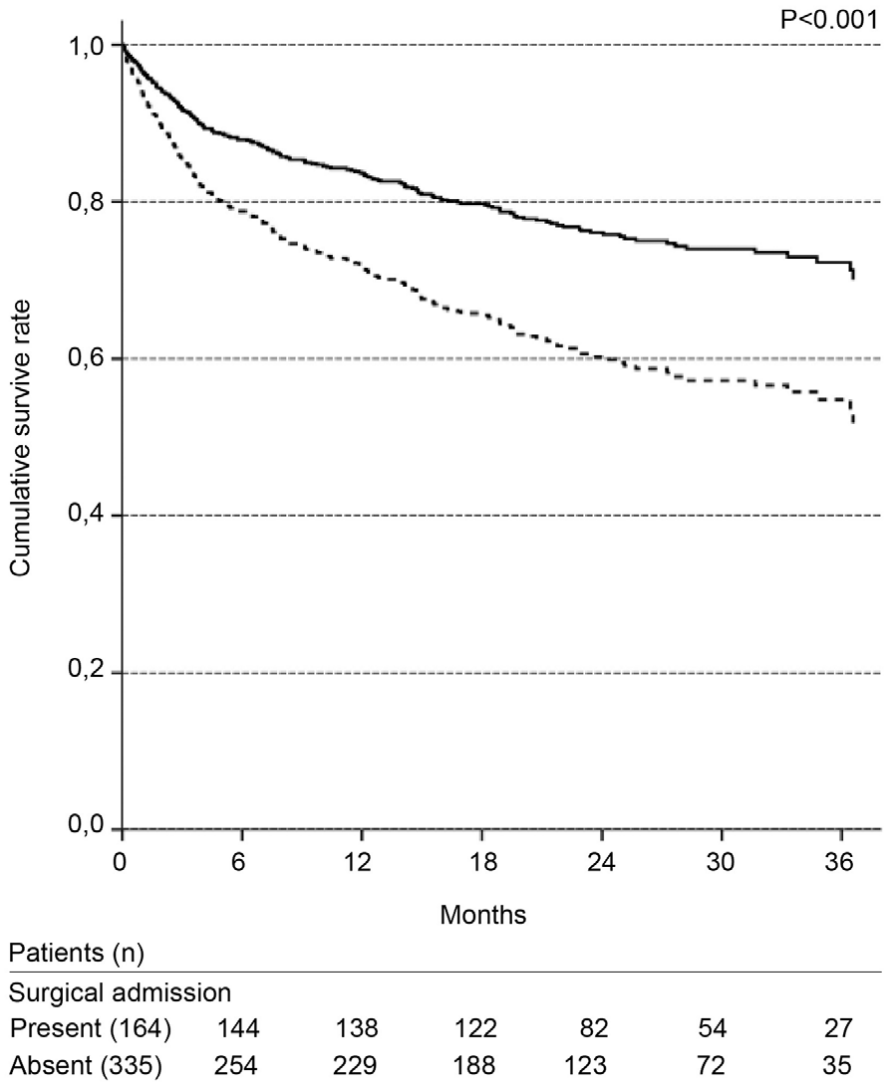
Cox survival curves according to type of ward admission. Solid line: surgical; dotted line: non-surgical. Adjusted for: gender; age; presence of cancer, heart failure, and presumed chronic kidney disease; nephrotoxic and septic etiologies.

**Figure 6:**
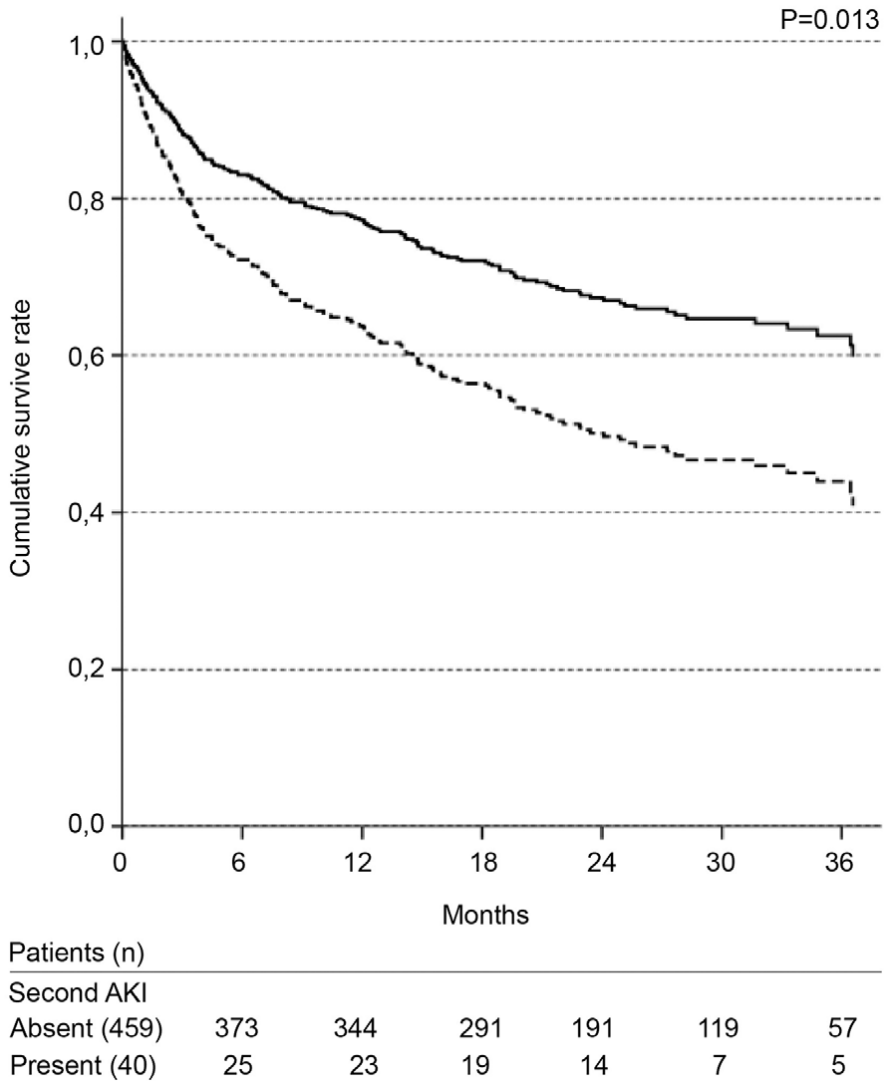
Cox survival curves according to the presence of a second AKI episode during the same hospitalization. Solid line: absence; dotted line: presence. Adjusted for: gender; age; presence of cancer, heart failure, and presumed chronic kidney disease; nephrotoxic and septic etiologies.

**Table 3:** Final Cox model.

	Reference value	HR	CI	*P*
*Khan Index*				
	Low risk	1		
	Medium risk	1.666	0.930–2.986	0.086
	High risk	2.540	1.382–4.669	0.003
*Presence of comorbidity*				
Chronic liver disease	Absence	1		
	Presence	1.932	1.159–3.220	0.012
*Type of admission*				
	Surgical	1		
	Non-surgical	1.851	1.309–2.619	<0.001
*Second AKI*				
	Absence	1		
	Presence	1.748	1.124–2.719	0.013

HR, Hazard ratio; CI, confidence interval; AKI, acute kidney injury.

Adjusted for: age, gender, presence of cancer, heart failure and presumed chronic kidney disease, septic and nephrotoxic etiologies.

### Statistical Analysis

Continuous variables are presented as the median and interquartile range (IQR) and are compared using the Mann-Whitney test. Categorical variables are presented as absolute values and percentages and are compared by Fisher or Chi-square tests as appropriate. The graphical survival curve of our patients was compared with that of the São Paulo population and matched for age. The survival curve of the São Paulo population was estimated until 2008 and drawn using data available from the “Brazilian Institute of Geography and Statistics” (IBGE) and SEADE (SEADE is the Portuguese abbreviation for the System for Data Analysis of the State of São Paulo) [31], [32]. Survival curves were estimated by the Kaplan-Meier method, and differences between curves were assessed with the log-rank test. A multiple survival analysis was performed using Cox proportional hazards regression analyses. The variables with *P*<0.15 in the univariate analysis were included in the first model: gender (reference: male); age (reference: <65 years); presence of chronic liver disease, heart failure, cancer and presumed CKD (reference: absence); Khan index (reference: low-risk); type of admission (reference: surgical); ICU admission (reference: no ICU); nephrotoxic or septic etiology (reference: absence); AKI severity (reference: stage I); need for mechanical ventilation (reference: no need) and second AKI (reference: absence). The proportional hazards test and plotted cumulative survival estimate after ln (-ln) transformation suggested that the hazards of these variables were proportional for the period analyzed. Statistical analysis was performed using Graph Pad Prism version 5.0 and SPSS version 15.0 for Windows.

**Table 4:** Causes of death of the patients with acute kidney injury and of the São Paulo population.

Age	Cause of death (ICD-10) (%)	
	AKI	Population of São Paulo City*
18–50 years	1. Neoplasms and Diseases of the circulatory system(18% each one)	1. External causes of morbidity and mortality (32%)
	2. Certain infectious and parasitic diseases (15%)	2. Diseases of the circulatory system (18%)
		3. Neoplasms (15%)
51–62 years	1. Diseases of the circulatory system (20%)	1. Diseases of the circulatory system (28%)
	2. Diseases of the respiratory system (18%)	2. Neoplasms (28%)
	3. Neoplasms (16%)	3. Diseases of the respiratory system (8%)
63–73 years	1. Neoplasms (22%)	1. Diseases of the circulatory system (40%)
	2. Diseases of the circulatory system (20%)	2. Neoplasms (26%)
	3. Diseases of the respiratory system and Diseases of thedigestive system (10% each one)	3. Diseases of the respiratory system (12%)
74–95 years	1. Diseases of the circulatory system (33%)	1. Diseases of the circulatory system (39%)
	2. Diseases of the respiratory system (17%)	2. Diseases of the respiratory system (18%)
	3. Neoplasms (14%)	3. Neoplasms (18%)

AKI, acute kidney injury; ICD-10, International classification of diseases, 10^th^ revision.

* Data from PRO-AIM.

The Research Ethics Committees of the Hospital das Clínicas and of the Municipal Health Department of São Paulo City approved the study (numbers: 0621/09 and 264/08, respectively) and waived informed consent because there was no intervention, the study was retrospective and the study used only a data bank, where confidentiality was guaranteed.

## Results

Of the 740 AKI patients who had been attended by the Nephrology Department of Hospital das Clínicas, São Paulo City, during the study period, and discharged alive, 193 were excluded for the reasons shown in Figure 1, and 547 were included. However, 40 patients were lost (7%) because we could not determine their situation at the index date in the end of the study. The remaining 507 patients were followed for 21 months (10–30 months). At the index date, 193 patients (38% of those studied) had died, and death occurred 5 months (2–14 months) after hospital discharge.

The comparison of the survival curve of AKI patients with that of the population of São Paulo City (Figure 2a) showed a much poorer survival for the AKI patients. The difference persisted even after age stratification (Figure 2b).

The patients’ characteristics are shown in Table 1.The median age of the patients was 63 years (51–73 years). Those who died during the study period (non-survivors) were older than survivors [68 years (53–76 years) and 61 years (48–78 years), respectively, P<0.0001] and had a higher Khan index. Among the 507 studied patients, only 26 (5%) had no comorbidity.

The characteristics of AKI are presented in Table 2. LOS was similar between the groups: 30 days (17–56 days) for the survivors and 28 days (13–62 days) for the non-survivors (P = 0.45). The length of the nephrological follow-up was also similar between the groups: 8 days (4–14) for the survivors and 7 days (4–13) for the non-survivors (P = 0.12).

For 49 patients (9.6%), we used the lowest SCr value achieved during hospitalization as the reference SCr. Although the reference GFR tended to be lower in the non-survivors than in the survivors [57 (35–75) and 61 mL/min/1.73 m^2^ (39–88), respectively, P<0.08], the presence of presumed CKD was similar in both groups. The maximum SCr value achieved during the AKI was similar in both groups: 3.5 mg/dL (2.7–4.9 mg/dL) in non-survivors and 3.8 mg/dL (2.8–5.2 mg/dL) in survivors (P = 0.27). The severity of the AKI was also similar between the groups. At hospital discharge, the SCr value was 1.4 mg/dL (1.0–2.0 mg/dL) in the survivors and 1.5 mg/dL (1.0–2.1 mg/dL) in the non-survivors (P = 0.18). At the time of hospital discharge, 252 patients (50%) presented complete renal recovery, and this rate was similar between the survivors and the non-survivors, as shown in Table 2.

ICU admission and the need for mechanical ventilation were not significant in the first Cox regression model tested. Due to the number of missing values in these variables, a second model was evaluated without these two variables. The significant variables in the final Cox model are presented in Table 3. The independent variables associated with long-term mortality were as follows: classification of high risk according to the Khan index, presence of chronic liver disease, non-surgical admission and presence of a second episode of AKI (Figures 3–6, Table 3).

### Causes of Death

We obtained the death certificate of 164 non-survivors (85%). The main causes of death were diseases of the circulatory system (28%) and neoplasms (20%). Among the 47 patients who died from diseases of the circulatory system, 28 (59%) had hypertension, 16 (34%) had diabetes, 22 (47%) had heart failure, 18 (38%) had coronary diseases, and 31 (66%) had presumed CKD before the AKI episode. Among the 33 patients who died from neoplasms, 26 (79%) already had the disease at the time of hospitalization for the AKI episode. Only 19 non-survivors (11%) had none of the comorbidities cited above, and their main causes of death were diseases of the digestive system (7 patients, 37%) and diseases of the circulatory system (4 patients, 21%).

The causes of death of the AKI patients are presented in Table 4 and are compared with the causes of death for the population of São Paulo City of a similar age. For this analysis, the age of the AKI patients was divided into quartiles. The frequency of the main causes of death of the AKI patients was similar to that of the general population except in the first age quartile, or 18–50 years. The causes of death among the youngest AKI patients were neoplasms and diseases of the circulatory system (18% each), while the population of São Paulo City in the same age range died mainly from external causes (32%).

The main causes of death of the patients who died up to 6 months after hospital discharge were diseases of the circulatory system and neoplasms (24% each). Otherwise, after this period only 15% of deaths were due to neoplasms.

It is important to note that the patients who suffered a second AKI episode died from the same diseases as those who had not, including diseases of the circulatory system, neoplasms and diseases of the respiratory system.

## Discussion

The present study showed that patients who suffered an AKI episode caused by presumed ATN, who were followed by nephrologists during hospitalization and who were discharged free from dialysis remains with a high mortality even after hospital discharge. This mortality was much higher than those of the population of São Paulo City, and also higher than those reported in other studies [3], [33]. One explanation could be that we included all deaths occurred after hospital discharge (median time to death was 5 months) and some studies excluded the deaths up to 90 days [3], [33], [34].

Since half of non-survivors died in the first 6 months after discharge, and neoplasm was a much higher cause of death than in those who died after this period, it is reasonable to suppose that the high early mortality observed occurred in the most severely diseased patients.

The present study highlights the importance of previous comorbidities in long-term mortality after AKI. It showed that patients classified as high risk by the Khan index, those with chronic liver failure, those who were admitted to non-surgical services and those who had a second episode of AKI had a very high risk of death after hospital discharge.

The role of previous comorbidities on long-term mortality was more clearly shown by the finding that the non-survivors died from diseases already present before the AKI episode and that their causes of death were similar to the population of São Paulo City in the same age range.

The Khan index was first described as an indicator of the risk of mortality for patients with end-stage renal disease (ESRD) and was later transposed to AKI patients [35]. Ali *et al* showed that the index was associated with 6-month mortality after AKI [28]. In the present study, the patients classified as high risk by the Khan index had a poorer survival rate than those classified as low risk. There was a trend (P = 0.08) suggesting that the patients classified as medium risk also had a poorer prognosis than the low risk patients.

In addition to its presence on the Khan index, the presence of chronic liver disease was an independent factor associated with long-term mortality. It has been reported that the occurrence of AKI is frequent in patients with chronic liver failure and that it increases morbidity and mortality [36]. Even for patients who had received a liver transplant, a reduced GFR before the transplant was an independent predictor of mortality 30 days and 2 years after the transplantation [37]. Deshpande *et al* propose that the combination of AKI and liver failure produces a “toxic milieu” that directly causes endothelial dysfunction affecting multiple organ systems, which contributes to the increased short- and long-term mortality of these patients [36]. Our findings emphasize the catastrophic role of the interaction between chronic liver disease and AKI.

Admission to non-surgical wards was an independent factor associated with long-term mortality that could not be explained by the presence of any of the variables analyzed. Other studies have also found a poorer survival among non-surgical admissions, and this finding has not yet been explained [1], [10].

The impact that a second episode of AKI has on in-hospital mortality has not been studied. In the present study, only 8% of the patients had a second episode of AKI during the same hospitalization; however, those who did have a second episode were at very high risk for long-term mortality. More studies are needed to evaluate the importance of a new AKI episode both on in-hospital mortality and on long-term mortality.

Data from animal studies have already shown that AKI can induce damage to other organs, such as the lungs [38] and heart [39]. Thus, each AKI episode may aggravate pre-existing dysfunctions of various organs, accelerating their decline and leading to death.

AKI may also aggravate previous kidney disease, accelerate the progression to ESRD and worsen survival rates. Experimental studies have shown that AKI induces permanent kidney damage [40]–[42]. However, human studies are controversial, mainly because many studies on long-term mortality exclude patients with CKD [1], [5]–[7], [13]. Some studies have shown that in-hospital mortality is lower in patients with AKI superimposed on CKD (AoCKD) when compared with patients with “pure” AKI [43], [44]. However, Ali *et al* showed that the 6-month mortality of patients with AoCKD was higher than those with AKI (62.5% and 49.8%, respectively, P = 0.038) [28]. Long-term mortality was not associated with pre-existing CKD in the present study. However, we must note that our percentage of patients with presumed CKD (51%) was much higher than those reported by Ali *et al* (15%). This difference may be because in the present study, all patients were seen by nephrologists, while in the Ali *et al* study, the nephrologists attended only 29% of the patients [28].

Another controversial point is whether AKI severity is a good predictor of long-term mortality, as it has been shown to be for in-hospital mortality [45]. Usually AKI severity can be evaluated by AKIN or RIFLE criteria. As there is no widely accepted AKI definition for retrospective research using databases, we defined AKI considering the adapted AKIN definition [46]. This definition uses the ratio between the higher SCr measured during hospitalization and the previous SCr to define and classify the AKI episode and has been used by other authors [3], [11], [12], [14]. In contrast to these studies, which have shown that this classification can also predict long-term mortality [3], [11], [12], [14], in the present study, AKI severity was not a risk factor for long-term mortality. One factor may explain this difference; as mentioned earlier, we only included patients followed by nephrologists. Ponce *et al* showed that AKI patients evaluated by nephrologists were more seriously ill than those not evaluated [47]. This finding may explain why 49% of our patients were classified as AKI severity III, while other authors, who also evaluated patients not attended by nephrologists, reported that 13% of patients were classified as AKI severity III [3]. Moreover, patient with pre-renal AKI were not evaluated in the present study, as those with less than two days of follow-up were excluded.

Some studies found that the absence of renal recovery was a risk factor for long-term mortality [3], [7], [13], but in the present study, renal recovery was not associated with long-term mortality. Similar to our findings, Loef *et al* did not find a difference in mortality 96 months after the AKI episode between those who recovered and those who did not show renal function recovery [48]. Conversely, Mehta *et al* showed that, 13 years after cardiac surgery, the survival curve of the patients who did not show renal function recovery was poorer than that of those who had recovered (P = 0.046) [13]. However, in the present study patients who were discharged on dialysis and also those with AKI not due to presumed ATN were excluded. These exclusions might explain this difference. It must be noted that the definition of renal recovery varies among studies, and it may be more appropriate to use a SCr measured after hospital discharge to define renal recovery [49].

The present study has some limitations. The lack of a control group, similar to the studied population but without AKI, does not allow us to establish a causal relationship between AKI and long-term mortality. Although we attempted to control for selection bias with statistical methods using multiple variables, our retrospective observational analysis is subject to bias from unmeasured factors. However, it is very difficult to find a matched control group. Wald *et al*, who studied 4066 AKI survivors, had to exclude 297 more seriously ill patients because they could not match these patients to an adequate control [34]. In the present study, we compared the long-term survival of AKI patients with the population of São Paulo City at the same age and found poorer survival among AKI patients. Although the general population is not an ideal control group, a similar approach was taken by Liaño *et al*, who compared the mortality of AKI patients with the mortality of the population of Madrid and found poorer survival among AKI patients [1].

The use of death certificates to address the cause of death is subject to bias, as it depends on the person who completed the certificate. In 2005, the World Health Organization (WHO) examined the quality of the death certificates in 115 countries and classified the Brazilian death certificates to be of medium quality, based on the percentage of incomplete data and ill-defined codes [50]. However, França *et al* analyzed the data in various regions of Brazil and found that the quality of the data was high in the southeast (where São Paulo City is located) [51]. Moreover, we observed that only 5 death certificates (3%) had an improperly defined code as the cause of death.

The present study only included patients followed by nephrologists, and thus, some patients with AKI may have not been evaluated (presumably those with less severe disease). Other important studies, primarily those based on the Program to Improve Care in Acute Renal Disease (PICARD), also included only patients attended by nephrologists [1], [44]. The results of the present study cannot be generalized. However, they point to the main importance of pre-existing comorbidities in patients with AKI due to presumed ATN who were discharged free from dialysis.

Our study also has several strengths. It was conducted in a developing country, where there is a lack of true epidemiologic studies on AKI [21], [22]. We used a globally accepted AKI definition that was based not on a calculated reference Scr but on a reported reference Scr [3], [11], [12], [14]. We lost only 40 patients (7%) after hospital discharge (Figure 1). We were also able to clearly characterize the AKI episode, which allowed us to adjust the analysis of outcomes for many important factors. To the best of our knowledge it was the first time that the causes of long-term mortality were carefully studied.

In conclusion, long-term mortality after an AKI episode due to presumed ATN is high and is more closely related to previous comorbidities than to the characteristics of the AKI. In our view, all survivors of AKI should have a medical follow-up after hospital discharge and all efforts should be made to control their comorbidities.

## Acknowledgments

Partial results were presented at World Congress of Nephrology 2009 and at Renal Week 2010.
